# The N-Terminal Cleavage of Chondromodulin-I in Growth-Plate Cartilage at the Hypertrophic and Calcified Zones during Bone Development

**DOI:** 10.1371/journal.pone.0094239

**Published:** 2014-04-07

**Authors:** Shigenori Miura, Jun Kondo, Aki Takimoto, Hiroko Sano-Takai, Long Guo, Chisa Shukunami, Hideyuki Tanaka, Yuji Hiraki

**Affiliations:** 1 Department of Cellular Differentiation, Institute for Frontier Medical Sciences, Kyoto University, Kyoto, Japan; 2 Research and Development Division, Science and Technology Research Center Inc., Mitsubishi Chemical Group, Kanagawa, Japan; 3 Department of Dental and Medical Biochemistry, Basic Life Sciences, Institute of Biomedical & Health Sciences, Hiroshima University, Hiroshima, Japan; University of Maryland School of Medicine, United States of America

## Abstract

Chondromodulin-I (ChM-I) is a 20–25 kDa anti-angiogenic glycoprotein in cartilage matrix. In the present study, we identified a novel 14-kDa species of ChM-I by immunoblotting, and purified it by immunoprecipitation with a newly raised monoclonal antibody against ChM-I. The N-terminal amino acid sequencing indicated that it was an N-terminal truncated form of ChM-I generated by the proteolytic cleavage at Asp^37^-Asp^38^. This 14-kDa ChM-I was shown by the modified Boyden chamber assay to have very little inhibitory activity on the VEGF-A-induced migration of vascular endothelial cells in contrast to the intact 20–25 kDa form of ChM-I (ID_50_ = 8 nM). Immunohistochemistry suggested that 20–25 kDa ChM-I was exclusively localized in the avascular zones, i.e. the resting, proliferating, and prehypertrophic zones, of the cartilaginous molds of developing long bone, whereas the 14-kDa form of ChM-I was found in hypertrophic and calcified zones. Immunoblotting demonstrated that mature growth-plate chondrocytes isolated from rat costal cartilage actively secrete ChM-I almost exclusively as the intact 20–25 kDa form into the medium in primary culture. Taken together, our results suggest that intact 20–25 kDa ChM-I is stored as a component of extracellular matrix in the avascular cartilage zones, but it is inactivated by a single N-terminal proteolytic cleavage in the hypertrophic zone of growth-plate cartilage.

## Introduction

Cartilage is highly resistant to vascularization and is known as a typical avascular tissue among tissues of mesenchymal origin except for hypertrophic and calcified cartilage, which is vascularized during endochondral bone formation [1], [2]. To assure the avascularity of cartilage, the presence of a specific angiogenesis inhibitor has been envisioned in cartilage ECM [2], [3], [4]. Based on this premise, we purified an inhibitory activity from the 1 M guanidine extracts of fetal bovine cartilage by monitoring its action on the growth and tube morphogenesis of cultured vascular endothelial cells to result in an identification of Chondromodulin-I (ChM-I) as a cartilage-drived angiognesis inhibitor [5], [6], [7]. It also inhibited VEGF-A-induced corneal angiogenesis and chemotactic migration of vascular endothelial cells [8], and is implicated in the maintenance of avascularity in cartilage, inner meniscus, heart valves, and some specific eye compartments such as iris-ciliary body, retina, and scleral compartments [7], [9], [10], [11].

**Figure 1:**
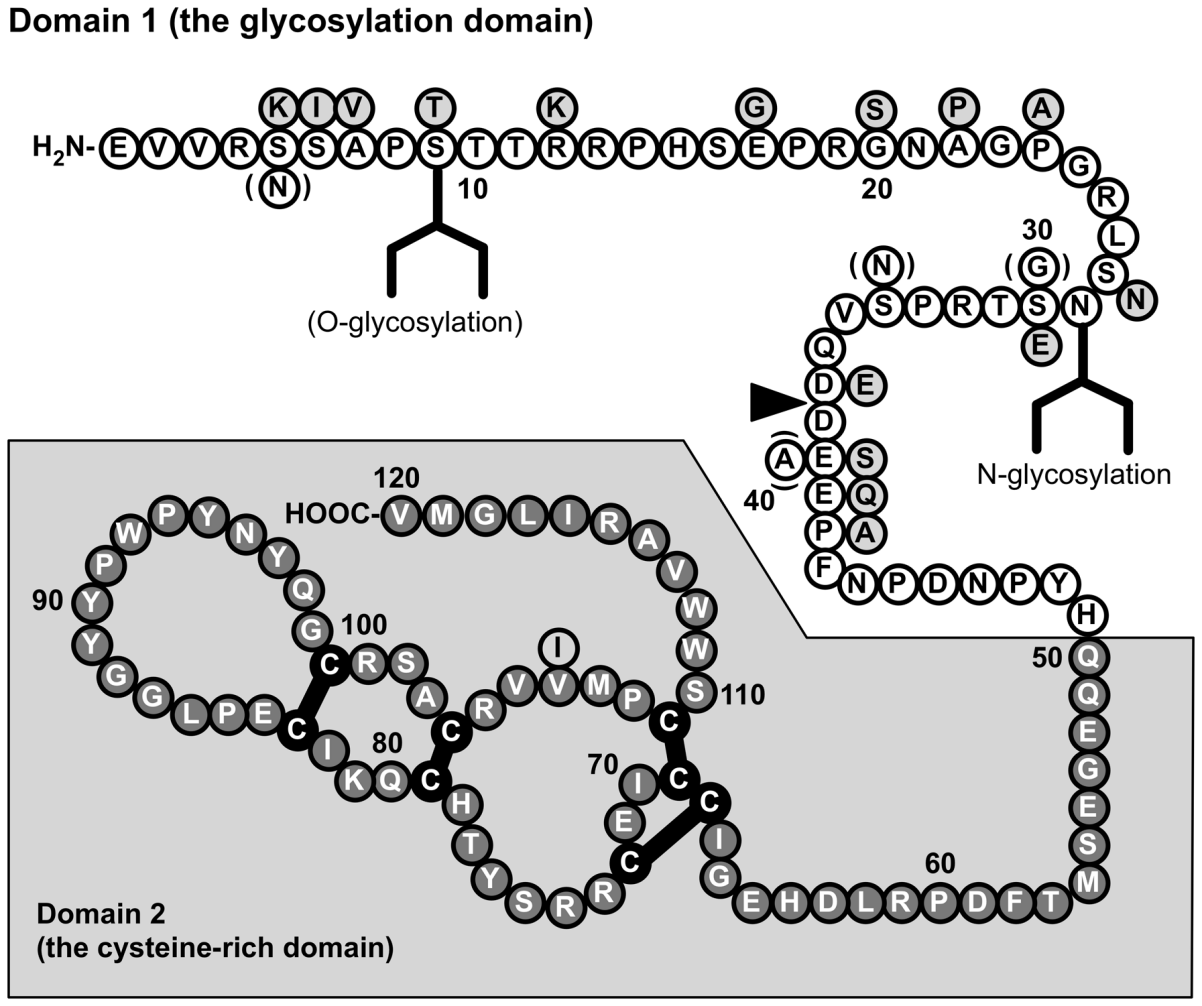
The amino acid sequence of rat chondromodulin-I (rChM-I). Light gray circles indicate amino acid residues that are not conserved in human ChM-I (hChM-I). Circles in parentheses indicate four amino acid residues that are not conserved in mouse (mChM-I). Four thick bars connecting a pair of Cys residues indicate the intramolecular disulfide bonds, whose arrangements are assumed to be identical with those determined for bChM-I purified from fetal bovine cartilage [27], [28]. The putative glycosylation sites are also indicated. The arrowhead indicates the determined cleavage site that gives rise to the 14-kDa form of ChM-I.

Human ChM-I and its murine counterparts are a single chain glycoprotein with 120 amino acid residues, which is recognized as a broad 20–25 kDa band in tissue extracts by immunoblotting [6], [7]. The ChM-I molecule is composed of two structurally distinct domains (Fig. 1): the N-terminal hydrophilic domain (Domain 1) containing the N- and O-linked oligosaccharide chains and the C-terminal hydrophobic domain (Domain 2) [12], [13]. The *in vitro* studies indicated that Domain 1 plays a critical role for maintaining the solution stability of ChM-I under physiological conditions [13], whereas Domain 2 is a stretch of 71 amino acid residues that is highly conserved among species [14]. Recently four disulfide bridges and a hydrophobic C-terminal tail (from Tryp^111^ to Val^120^) in Domain 2 were shown to be critical for the anti-angiogenic activity of ChM-I [14].

Antibodies against a synthetic peptide corresponding to the N-terminal hydrophilic sequence have been successfully used for the immunological detection of ChM-I to demonstrate that mature glycosylated 20–25 kDa ChM-I was specifically localized to cartilage ECM in the *Chm1* transcript-positive resting, proliferating, and prehypertrophic zones of growth-plate in developing long bone. No immunoreactivity was detected in the hypertrophic and calcified zones where *Chm1* mRNA was negative by *in situ* hybridization [5], [7], [15]. In the meantime, several monoclonal antibodies against glycosylated recombinant ChM-I were raised. Using these antibodies, we identified a novel 14-kDa ChM-I immunoreactive band on SDS-PAGE in cartilage extracts. In the presnt study, we report isolation of this short form of ChM-I from rat cartilage extracts by immunoprecipitation and determination of its N-terminal amino acid sequence. Differential localization of 14-kDa and 20–25 kDa ChM-I in growth-plate cartilage of developing long bone is also studied by immunohistochemistry.

## Results

### Identification of 14-kDa fragment of ChM-I in cartilage extracts

**Figure 2:**
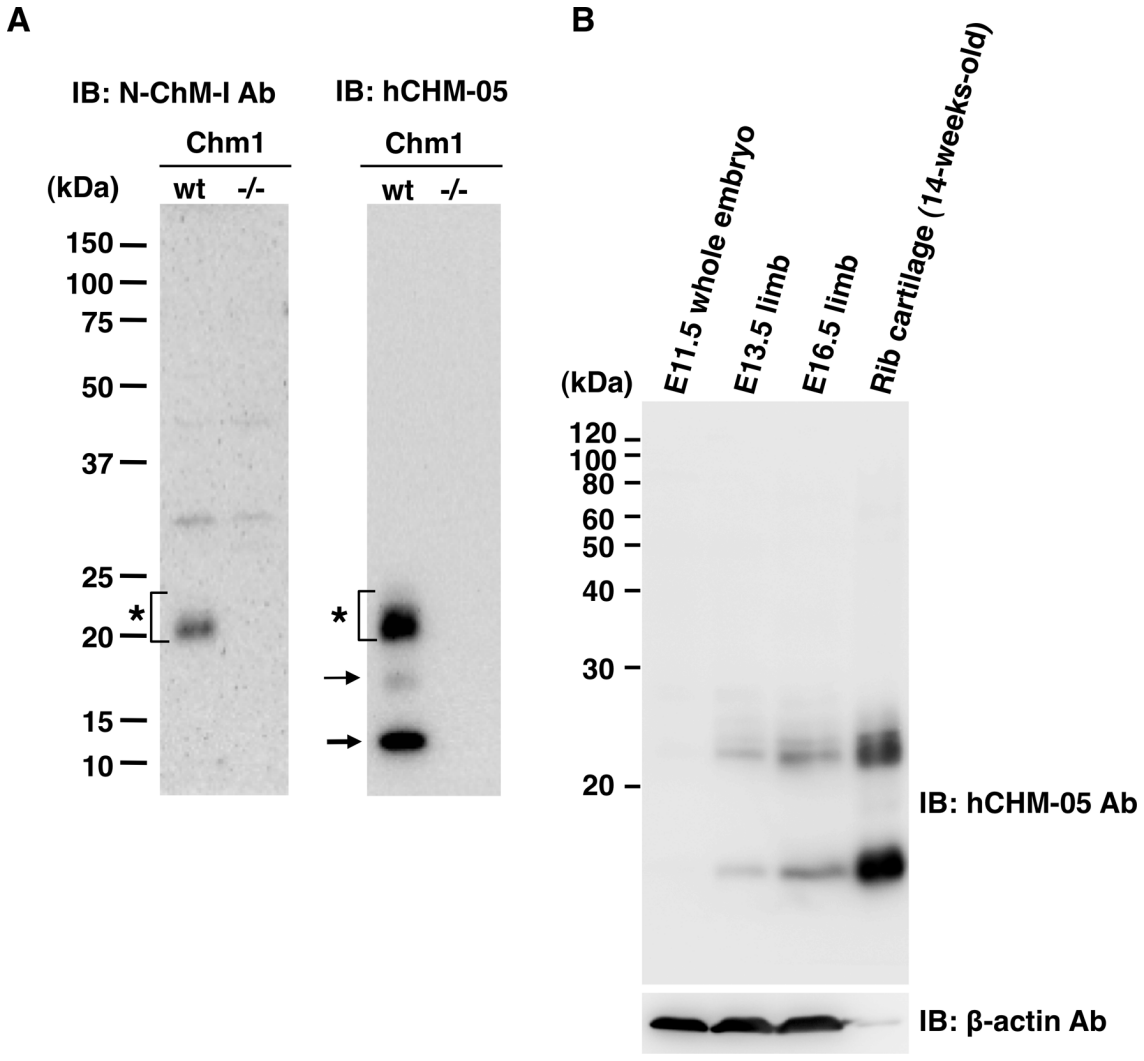
Identification by immunoblotting of a 14-kDa fragment of ChM-I in mouse cartilage extracts. (A) Cartilage extracts of 14-week-old male mice were prepared from ribs of wild-type (wt) and *Chm1* (-/-) null mice, and analyzed by immunoblotting with N-ChM-I Ab or hCHM-05 ChM-I MoAb. N-ChM-I Ab recognizes mouse 22-kDa glycosylated mature ChM-I (asterisks) as a broad immunoreactive band, whereas hCHM-05 ChM-I MoAb indicated the presence of a 14-kDa band (thick arrow) as well as a faint 17-kDa band (arrow). None of these bands were detected in the extracts from cartilage of *Chm1* (-/-) null mice. (B) Mouse tissue extracts were prepared from E11.5 whole embryos, and limbs dissected out at E13.5 and E16.5 as well as rib cartilage from 14-week-old male mice, and analyzed by immunoblotting with hCHM-05 ChM-I MoAb or β-actin antibody as a loading control. Note that the loading amount of rib cartilage extracts was reduced to 2.5 μg protein to give a quantitative range of signals in the same blot, while 10 μg protein was loaded per each lane for embryonic tissue extracts.

The synthetic N-terminal domain peptide of ChM-I, which corresponds to the rat sequence from Pro^8^ to Pro^33^ as well as the human counterpart (Fig. 1), has been utilized in raising an anti-N-terminal peptide antibody (N-ChM-I Ab) for detecting ChM-I by Western blotting and immunohistochemistry [6], [7]. This N-ChM-I Ab also recognizes mature ChM-I in urea extracts of mouse cartilage as a single 20–25 kDa broad band after SDS-PAGE (Fig. 2A). In the extracts from *Chm1* null mice [16], no immunoreactive protein was detected in the corresponding region of molecular size (Fig. 2A).

We recently succeeded in raising several clones of anti-ChM-I monoclonal antibody (ChM-I MoAb) against glycosylated recombinant human ChM-I (G-rhChM-I) [6], [8]. Many of these including the clone hCHM-05, which was used in the present study, reacted well with ChM-I from a variety of mammalian species upon immunoblotting. Interestingly these MoAb clones clearly visualized the presence of a novel 14-kDa protein band on immunoblotting as well as a 17-kDa faint band in cartilage extracts prepared from wild-type mice (Fig. 2A, the right panel). No immunoreactive band was detected in cartilage extracts prepared from *Chm1* null mice, indicating that these protein bands are likely to be derived from ChM-I.

As shown in Fig. 2B, this 14-kDa protein band was absent in the extracts of whole mouse embryos prior to the formation of cartilaginous bone primordia by E11.5, but became detectable soon after the formation of cartilage in the body concomitantly with the appearance of the 20–25 kDa ChM-I band. Semiquantitative analysis of the immunoreactive bands indicated that the relative intensity of the 14-kDa band over the 20–25 kDa ChM-I band increased with development and growth: 1.85±0.25 at E13.5, 2.42±0.38 at E16.5 and 2.97±0.01 at 14 weeks after birth (the values are the means ± s.d. of three independent experiments).

### Immunoprecipitation of 14-kDa ChM-I from rat costal cartilage

**Figure 3:**
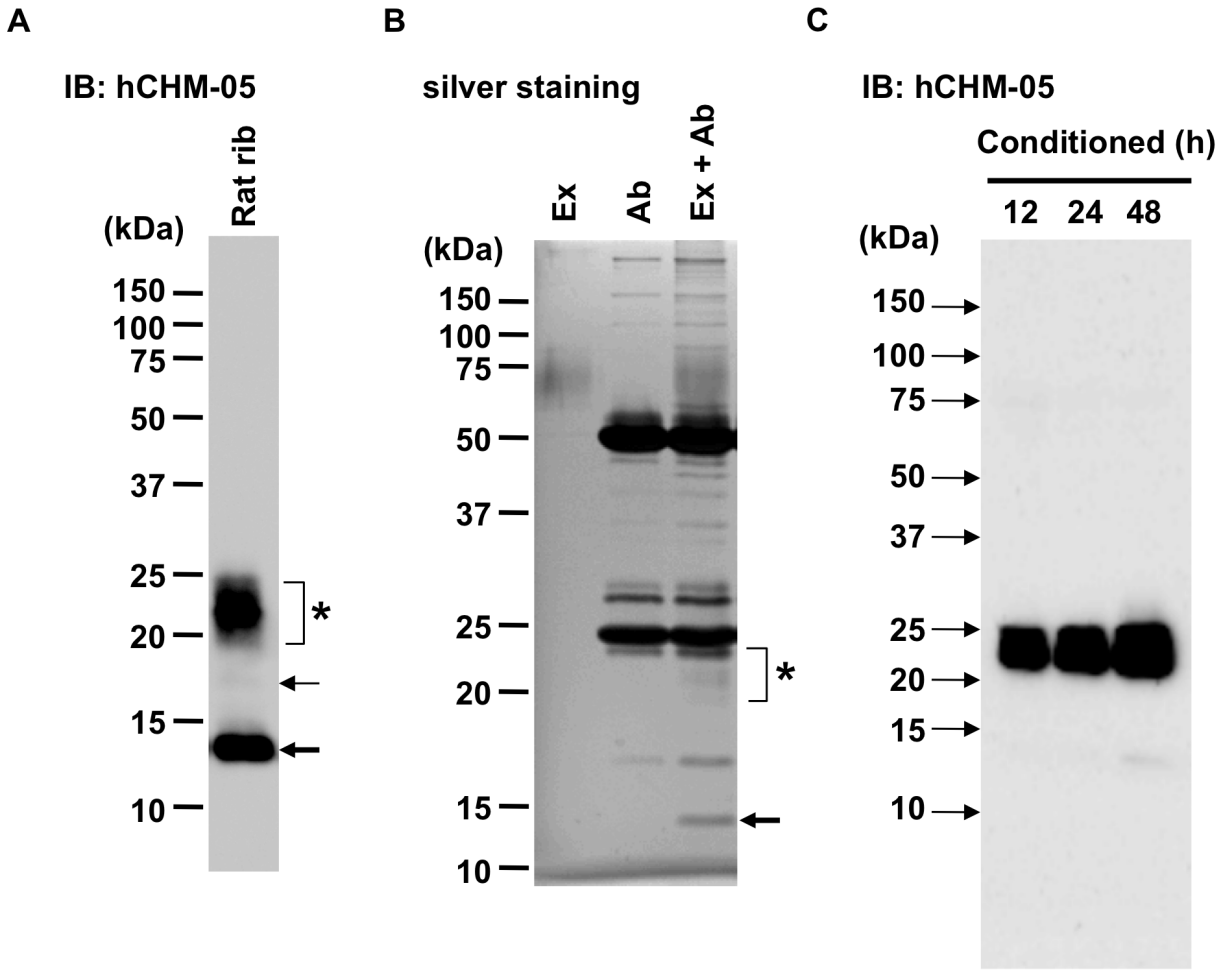
Identification and immunoprecipitation of rat 14-kDa ChM-I. (A) 8 M urea extracts were prepared from male rat ribs (4-week-old) and subjected to immunoblotting with hCHM-05 ChM-I MoAb. Rat 20–25 kDa glycosylated ChM-I (asterisk) was detected as a broad band as well as a 14-kDa band (thick arrow) and a faint 17-kDa band (arrow). (B) Rat ChM-I extracted in 8 M urea buffer was immunoprecipitated with hCHM-05. Immunoprecipitates were resolved by SDS-PAGE and detected by silver staining. For the specification of immunoprecipitated bands (Ex + Ab), cartilage extracts without the antibody (Ex) or 8 M urea buffer with the antibody (Ab) were similarly processed during the immmunoprecipitation. The asterisk and the arrow indicate 20–25 kDa and 14-kDa ChM-I, respectively. (C) Primary cultures of rat costal chondrocytes were cultured to confluence for 3 days and then conditioned in DMEM/F12 medium in the presence of 10% FBS for the indicated periods of time. The collected conditioned medium was concentrated and subjected to immunoblotting with hCHM-05 ChM-I MoAb.

The hCHM-05 ChM-I MoAb recognized 14-kDa immunoreactive protein similarly in the urea extracts of rat costal cartilage as a major band in addition to the 20–25 kDa mature intact form of rat ChM-I (Fig. 3A). The 17-kDa protein band was also discernible as a faint band. We then decided to isolate these proteins from rat cartilage by immunoprecipitation with the hCHM-05 ChM-I MoAb in a large-scale. Thus 90 mg of rat costal cartilage was homogenized and extracted in 8 M urea as described in the “Materials and Methods” section. Immunoprecipitation and the following separation of proteins by SDS-PAGE yielded about 200 ng of the 14-kDa protein (as indicated by the arrow in Fig. 3B) in addition to the 20–25 kDa ChM-I. The N-terminal amino acid sequence of this 14-kDa protein was determined to be the rat ChM-I sequence starting from Asp^38^ (Fig. 1), indicating that it was a proteolytic product of ChM-I (14-kDa ΔN-ChM-I) generated by the cleavage at the site of a diacidic sequence (Asp^37^-Asp^38^). Due to a very low abundance, 17-kDa protein was failed to be isolated.

### Secretion of the mature 20–25 kDa form of ChM-I by cultured growth-plate chondrocytes

Non-calcified mature growth-plate chondrocytes were isolated from costal cartilage of 3-weeks-old Wistar rats and grown to confluence for 3 days in the presence of 10% fetal bovine serum (FBS). Under the present culture conditions, chondrocytes rarely undergo terminal differentiation to be hypertrophic cells. Almost all of the cells express mature non-hypertrophic phenotype of chondrocytes [17]. After the cultures reached confluence, the culture medium was replaced by the fresh medium containing 10% FBS, and conditioned for another 12, 24, or 48 h. The collected conditioned-medium was analyzed by immunoblotting with hCHM-05 ChM-I MoAb, demonstrating that cultured chondrocytes actively secreted ChM-I into culture medium over the time (Fig. 3C). Interestingly these primary cultured chondrocytes almost exclusively secreted the intact 20–25 kDa form of ChM-I into medium. No 14-kDa immunoreactive band was detected in the medium conditioned for 12 or 24 h. After 48 h of conditioning, the 14-kDa band became only faintly discernible.

### Bioactivity of 14-kDa ChM-I *in vitro*

**Figure 4:**
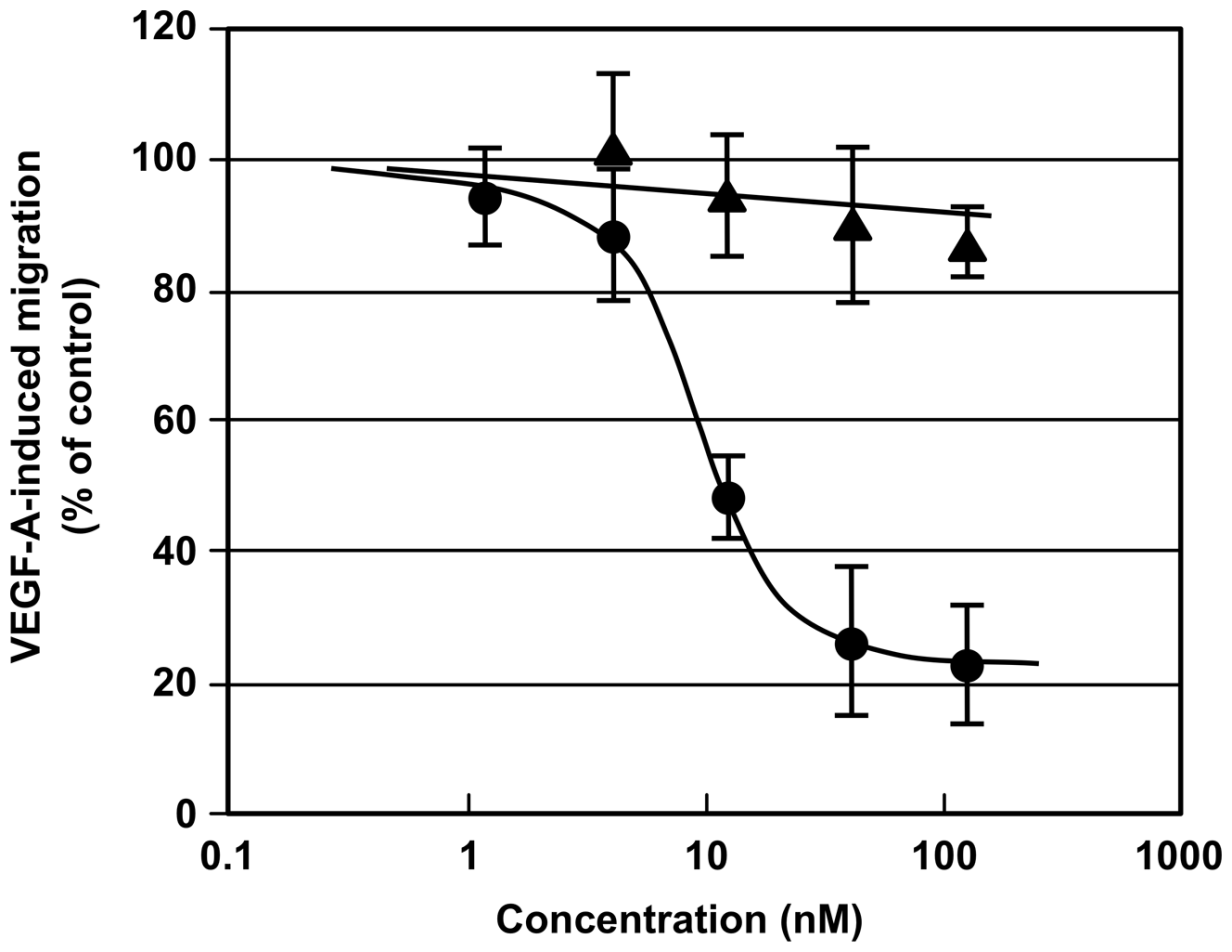
Effects of 14-kDa ΔN-rhChM-I on the VEGF-A-induced chemotactic migration of HUVECs. Biological activity of G-rhChM-I and ΔN-rhChM-I was assessed by a modified Boyden chamber assay [8]. Serum-starved HUVECs (7×10^4^ cells) were preincubated for 30 min with G-rhChM-I (•) or ΔN-rhChM-I (▴), and seeded onto vitronectin-coated cell culture inserts in serum-free medium. Chemotactic migration of HUVECs was induced by the addition of VEGF-A (20 ng/ml) in the lower chamber, and the cells were allowed to migrate for 4 h toward VEGF-A. The number of cells that had migrated to the bottom surface of the insert was counted. Values are the percentages of migrated cell numbers in the control culture that was stimulated by VEGF-A, and are the means ± SD of a triplicate assay. The data are representative of three independent experiments with similar results.

The diacidic sequence motif in Domain 1 is conserved among mammalian species, e.g. Glu^37^-Asp^38^ in human ChM-I and Asp^37^-Asp^38^ in murine ChM-I (Fig. 1). We previously reported a preparation of 14-kDa rhChM-I lacking the N-terminal 37 amino acids (ΔN-rhChM-I) by digestion with V8 proteinase [13]. Thus we could examine bioactivity of 14-kDa ΔN-ChM-I using ΔN-rhChM-I as a human recombinant counterpart by comparing its inhibitory activity with intact G-rhChM-I on the VEGF-A-induced migration of human umbilical vein endothelial cells (HUVECs) in a modified Boyden chamber assay [8], [14]. Intact 20–25 kDa G-rhChM-I exhibited a potent inhibitory action on the migration of HUVECs stimulated by an optimal dose (20 ng/ml) of VEGF-A (Fig. 4) [8]. Its half maximal inhibitory dose (ID_50_) was about 8 nM. In contrast, 14-kDa ΔN-rhChM-I showed only little inhibitory effect on the migration of HUVECs, indicating that intact 20–25 kDa ChM-I can be substantially inactivated by the cleavage of the N-terminal 37 amino acid residues. Due to a limited solubility of ΔN-rhChM-I [13], we could not carry out this migration assay at doses higher than 100 nM. Thus the determination of the ID_50_ value for 14-kDa ΔN-ChM-I was not possible.

### Differential distribution of 14-kDa ΔN-ChM-I and intact 20–25 kDa ChM-I in cartilaginous developing bone

**Figure 5:**
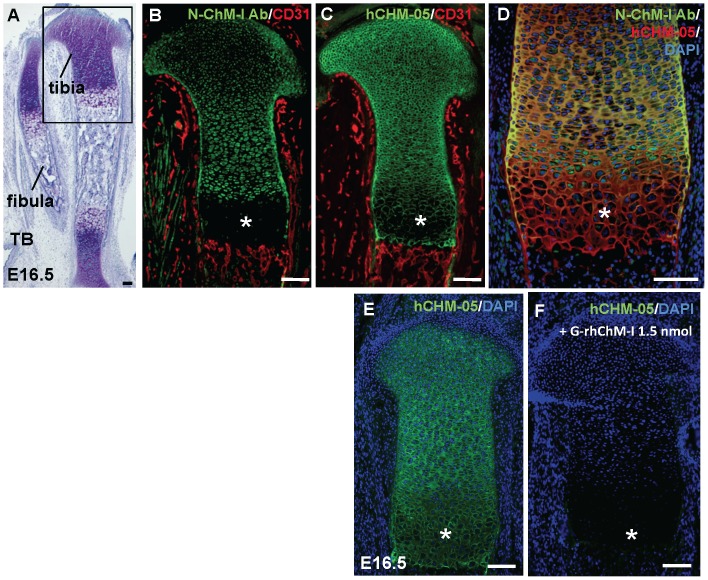
Differential distribution of 14-kDa ΔN-ChM-I and 20–25 kDa ChM-I in mouse developing bone at E16.5. Frozen sections were prepared from legs of an E16.5 mouse embryo. (A) Toluidine blue (TB) staining of the sagittal section of tibia and fibula. (B, C) Immunofluorescent views of the boxed area in panel A. Double immunofluorescent staining of the sagittal section of proximal tibia with CD31 (red) and N-ChM-I Ab (green; B) or hCHM-05 ChM-I MoAb (green; C). (D) Double immunofluorescent staining of the sagittal section of proximal tibia with N-ChM-I Ab (green) and hCHM-05 ChM-I MoAb (red). (E, F) Absorption test for hCHM-05 ChM-I MoAb is shown. The semiserial sections of tibia was immunostained with hCHM-05 ChM-I MoAb (green) preincubated with BSA (E) or G-rhChM-I (F). In panels D, E, and F, the nuclei were counterstained with DAPI (blue). Asterisks in the panels B-D indicate hypertrophic cartilage zone. Scale bars, 100 μm.

Taking advantage of N-ChM-I Ab and hCHM-05 MoAb, we attempted to study the localization of 14-kDa ΔN-ChM-I in mouse developing long bone immunohistochemically. Frozen sections were prepared from the legs of E16.5 mouse embryo, in which vascular invasion occurred in the primary ossification center and replacement of calcified cartilage into trabecular bone were in progress (Fig. 5A). N-ChM-I Ab visualized the localization of intact 20–25 kDa ChM-I in the resting, proliferating, and prehypertrophic zones of epiphyseal cartilage in bone primordia (Fig. 5B). The distribution of N-ChM-I Ab immunoreactivity apparently overlapped with that of *Chm1* mRNA as reported previously [5], [6], [18]. No immunostaining could be observed in the hypertrophic and calcified cartilage zones (Fig. 5B). The CD31-positive vasculature was found throughout the soft tissues, but clearly excluded from epiphyseal cartilage. In the primary ossification center, CD31-positive vasculature invaded calcified cartilage to make the bone forming front beneath the hypertrophic/calcified cartilage zone. In contrast, hCHM-05 ChM-I MoAb, which reacts with both 14-kDa ΔN-ChM-I and 20–25 kDa ChM-I, displayed immunoreactivity all through epiphyseal cartilage including the hypertrophic and calcified cartilage zones (Fig. 5C). The double immunofluorescent study demonstrated that ECM in the hypertrophic and calcified cartilage was only immunoreactive to hCHM-05 ChM-I MoAb, but not to N-ChM-I Ab (Fig. 5D), indicating that it contains only 14-kDa ΔN-ChM-I (Fig. 5D). Cartilage ECM stained yellowish is indicative of the presence of the intact 20–25 kDa ChM-I in the resting, proliferating, and prehypertrophic cartilage zones (Fig. 5D). The specificity of hCHM-05 MoAb was confirmed by an absorption test using G-rhChM-I as a competitor (Fig. 5E), while the specificity of N-ChM-I Ab was previously confirmed elsewhere [7].

**Figure 6:**
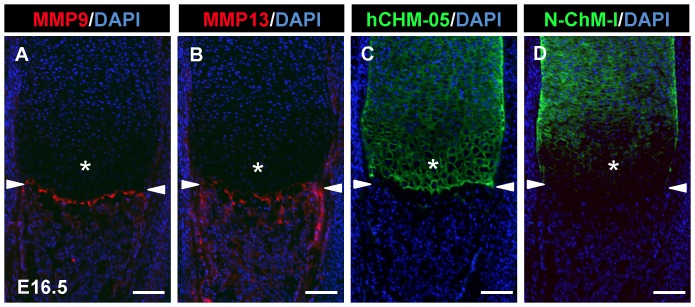
Localization of MMP9 and MMP13 in mouse developing bone at E16.5. Frozen sections were prepared from legs of an E16.5 mouse embryo. Serial sections of proximal tibia were stained with MMP9 antibody (red; A), MMP13 antibody (red; B), hCHM-05 ChM-I MoAb (green; C) or N-ChM-I Ab (green; D). The nuclei were counterstained with DAPI (blue). Asterisks indicate the hypertrophic cartilage zone. Arrowheads indicate the boundary between cartilage and the invading front of vasculature. Scale bars, 100 μm.

Several MMPs such as MMP9 and MMP13 are involved in the process of endochondral bone formation mainly at the step of vascular invasion [19], [20], [21], [22]. Thus, we compared the localization of these enzymes with that of intact 20–25 kDa ChM-I and 14-kDa ΔN-ChM-I. Immunofluorescent staining of serial sections of developing tibia at E16.6 was carried out (Fig. 6). The MMP9 immunoreactivity was localized in vascular endothelial cells invading the late hypertrophic/calcified cartilage zone in the primary ossification center (Fig. 6A), whereas the strong MMP13 immunoreactivity was associated with cartilage ECM at the interface of hypertrophic/calcified cartilage and invading blood vessels as well as cartilage remnants in the mineralizing trabecular bone (Fig. 6B). The localization patterns of these enzymes were quite distinct from that of hCHM-05 MoAb (Fig. 6C) and N-ChM-I Ab (Fig. 6D) as well.

## Discussion

In the present study, we described for the first time the occurrence of the N-terminal deleted form of ChM-I (14-kDa ΔN-ChM-I) in cartilage. The cleavage site was determined to be the site of a diacidic amino acid sequence (Asp^37^-Asp^38^) in Domain 1. This N-terminal cleavage results in the removal of most of the glycosylated hydrophilic Domain 1, which contains two putative glycosylation sites. Glycosylation of ChM-I was shown to be important for the maintenance of solution stability of ChM-I in physiological aqueous environment [13].

The 14-kDa form of ChM-I thus generated is supposed to be captured in cartilage ECM as an even firmly associated form due to the increased hydrophobicity, leading to its sequestration from the surrounding vascular tissues. Even though it contains the disulfide-bridged structure (Cys^83^-Cys^99^) and the C-terminal tail (Trp^110^-Val^120^), both of which are the critical structural elements required for the anti-angiogenic activity of ChM-I [14], the anti-migratory activity of 14-kDa ChM-I is hardly detectable by Boyden chamber assay probably due to its poorly-soluble nature and inability of taking active conformation to interact with cultured HUVECs. Non-glycosylated recombinant human ChM-I (NG-rhChM-I) was apparently less soluble in aqueous solution with physiological salt concentrations such as phosphate-buffered saline (PBS) or cell culture media than in distilled water. The measurement of CD (circular dichroism) spectra showed that the α-helical structure of bioactive glycosylated recombinant human ChM-I (G-rhChM-I) is not maintained in NG-rhChM-I, as reported previously [13]. Recombinant human 14-kDa ΔN-ChM-I, which was prepared from NG-rhChM-I by the limited proteolysis with V8 protease, was basically insoluble in PBS [13], indicating that 20–25 kDa ChM-I stored in cartilage ECM can be substantively inactivated by the N-terminal cleavage of the glycosylated portion.

Double immunofluorescent staining revealed in the present study that ECM at the hypertrophic and calcified zone of cartilage contains no intact 20–25-kDa ChM-I, but contains only the short form of ChM-I, i.e. 14-kDa ΔN-ChM-I. This short form of ChM-I was widely distributed in these terminally differentiated cartilage zones. Taking the fact into account that mature growth-plate chondrocytes secrete intact 20–25 kDa ChM-I in primary culture, it is reasonable to assume that ChM-I is first secreted out in ECM as an intact 20–25 kDa form, and then the N-terminal cleavage of ChM-I may take place somewhere in the extracellular space of cartilage by some unknown mechanisms. As a result, the rest of the molecule containing hydrophobic domain must be left in ECM at the hypertrophic and calcified cartilage zones where chondrocytes have already ceased to supply newly synthesized intact 20–25 kDa ChM-I into ECM [5], [6], [18], [23].

Occurrence of 14-kDa ΔN-ChM-I in hypertrophic and calcified cartilage may indicate that the N-terminal cleavage event of ChM-I is a quick deactivation response of cartilage to wipe out its anti-angiogenic property prior to vascular invasion and bone formation. Although it is shown that the relative ratio of 14-kDa ΔN-ChM-I over mature 20–25 kDa ChM-I seems to increase in association with development and rib growth plate ossification, the physiological significance of this event *in vivo* is obscure at this moment. As reported previously [24], cartilaginous bone primordia are wrapped up by the perichondrial anti-angiogenic barrier during development. This robust perichondrial barrier serves as a primary gate for vascular invasion and endochondral bone formation. Therefore, the withdrawal of ChM-I in the cartilage core of bone primordia by a simple gene knockout technology seems not effective for probing the physiological role of ChM-I and its N-terminal cleavage.

The actions of MMP9 and MMP13 are implicated in the massive degradation and remodeling of cartilage ECM during vascular invasion into cartilage of bone primordia. Actually replacement of cartilage into bone was significantly delayed in either *Mmp9* or *Mmp13* null mice [20], [21], [22]. The skeletal phenotypes of mice are further pronounced in MMP9 and MMP13 double knockout mice. In contrast to a wide distribution of 14-kDa ΔN-ChM-I in ECM of hypertrophic cartilage, the localization of MMP9 was almost exclusively confined to invading vascular endothelial cells, while MMP13 was found only in ECM of calcified cartilage at the interface of cartilage and invading vasculature. Abundant immunoreactivity against MMP13 was also shown in cartilage remnants in primary ossification center. These results indicated that these enzymes are unlikely to be responsible for generation of 14-kDa ΔN-ChM-I in cartilage. Actually our preliminary studies using G-rhChM-I as a substrate indicated that none of them could generate 14-kDa ΔN-ChM-I *in vitro* (data not shown). Biochemical analysis is currently underway to find out a specific ChM-I-cleaving endoprotease activity responsible for the 14-kDa ΔN-ChM-I generation in cartilage.

## Materials and Methods

### Materials

G-rhChM-I was expressed in Chinese hamster ovary (CHO) cells and purified from culture supernatants as described previously [6]. NG-rhChM-I was expressed in E. coli BL21 and purified as described [13]. The disulfide pairings were confirmed by trypsin digestion and peptide mapping of disulfide-bridged G-rhChM-I and NG-rhChM-I [6], [13]. The N-terminal deleted 14-kDa rhChM-I (ΔN-rhChM-I) was prepared by incubation of NG-rhChM-I with V8 protease (Pierce, Rockford, IL), and the resultant products were then purified by Butyl-Toyopearl 650 M (Tosoh, Tokyo, Japan) and reversed-phase HPLC [13]. Recombinant human vascular endothelial growth factor-A_165_ (VEGF-A_165_) and human vitronectin were purchased from R&D Systems (Minneapolis, MN) and BD Biosciences (Bedford, MA), respectively. Other chemicals were purchased from Sigma (St Louis, MO).

### Antibodies

A rabbit polyclonal anti-N-ChM-I antibody (N-ChM-I Ab) was raised against a synthetic oligopeptide (NH_2_-PSTTRRPHSEPRGNAGPGRLSNRTRP-CO_2_H) corresponding to the amino acids 8-33 of rat ChM-I [7]. A mouse monoclonal anti-ChM-I antibody (ChM-I MoAb) was raised in mice by immunizing recombinant human ChM-I expressed in 293-F cells [8]. The purified ChM-I MoAb (clone: hCHM-05) is commercially available from Cosmo Bio Co., Ltd. (Tokyo, Japan). Anti-CD31 antibody was obtained from BD PharMingen (San Diego, CA). Anti-MMP9 antibody was obtained from Santa Cruz Biotechnology (Dallas, TX). Anti-MMP13 antibody was from Merck Millipore (Billerica, MA). Anti-β-actin antibody (AC-15) was from Sigma.

### Animals

C57BL/6 mice and Wistar/ST rats were purchased from SHIMIZU laboratory supplies (Kyoto, Japan). *Chm1* knockout (KO) mice bred on C57BL/6 background were generated and maintained as described previously [16], [25]. All experiments were approved by the Animal Care Committee of the Institute for Frontier Medical Sciences, Kyoto University and conformed to the institutional guidelines for the study of vertebrates.

### Cell Culture

HUVECs (Lonza, Walkersville, MD) at passages 4–7 were grown to become subconfluent in endothelial cell growth medium (EGM-2 BulletKit; Lonza) and used in the migration assay [8]. Rat chondrocytes were isolated from costal cartilage of 3 week-old male Wistar rats [26]. Briefly, minced cartilage was incubated with 0.1% ethylenediamine tetraacetic acid (EDTA) at 37°C for 20 min and digested with 0.15% trypsin (Difco, Lawrence, KS) at 37°C for 1 h and 0.1% collagenase at 37°C for 3 h. Chondrocytes (5×10^5^ cells/well) were grown to confluence for 3 days in a 6-multiwell plate in a 1∶1 mixture of Dulbecco's modified Eagle's medium and Ham's F-12 medium (DMEM/F12 medium; Asahi Techno Glass, Funabashi, Japan) containing 10% FBS (HANA-NESCO BIO CORP., Tokyo, Japan). Then the culture medium was replaced by the fresh DMEM/F12 medium containing 10% FBS, and conditioned for another 12 h to 48 h. The collected conditioned medium was subjected to immunoblotting.

### Immunoblotting analysis

For preparation of mouse embryonic tissue extracts, whole embryos or limbs were dissected from mouse embryos (E11.5, E13.5, and E16.5) and homogenized in 8 M urea extraction buffer [50 mM Tris-HCl (pH 8.0) and 1 mM EDTA, 20× volume of the wet weight of tissue]. The homogenates were then incubated at 4°C for 1 h with gentle rocking and centrifuged to remove insoluble debris. Supernatants were finally concentrated three-fold by ultrafiltration (Nanosep Centrifual Devices, MWCO = 10 kDa, PALL Life Sciences, Ann Arbor, MI). For costal cartilage extracts, tissues were dissected from 14-week-old male mice (including *Chm1* KO mice) and 4-week-old male Wistar/SD rats. Dissected cartilage tissues were frozen in liquid nitrogen, crashed by Cryopress (CP-100, Microtec Nition, Chiba, Japan), homogenized in 8 M urea extraction buffer, and incubated at 4°C for 2 h. The resulting tissue extracts were centrifuged, and protein concentration of the supernatant was determined by BCA Protein Assay Kit (Thermo SCIENTIFIC, Rockford, IL). Ten microgram of protein was loaded per each lane unless otherwise indicated. All of the tissue samples were mixed with equal volume of 2× SDS sample buffer and boiled for 5 min before loading. Rat cultured chondrocytes were rinsed with DMEM/F12 twice and then solubilized by 1× SDS sample buffer. Rat chondrocyte conditioned media (2 ml) containing 10% FBS were cleared by centrifugation, diluted with equal volume of sterile water and incubated with Toyopearl Butyl-650 resin (TOSOH, Tokyo, Japan) for 2 h at room temperature. The bound protein was eluted with 70% ethanol, and the eluates were dried by evaporation. Dried samples were dissolved in 40 μl of 1× SDS sample buffer. Samples were boiled for 5 min and separated by SDS-PAGE in a 15% gel and transferred onto nitrocellulose membrane (Bio-Rad, Hercules, CA). The membranes were preincubated with 3.2% skim milk in Tris-buffered saline (TBS) for 30 min and incubated with hCHM-05 ChM-I MoAb (1∶4000), N-ChM-I Ab (1∶3000) at 4°C overnight. Blots were washed with TBS containing 0.05% Tween 20, and incubated with horseradish peroxidase-conjugated secondary antibody (GE Healthcare Bio-Sciences). Detection of antibodies was performed using the ECL Western Blotting Detection Reagents (GE Healthcare Bio-Sciences). Immunoblot images were captured by ImageQuant LAS 4000 Mini (GE Healthcare Life Sciences). The 14-kDa ChM-I to mature 20–25 kDa ChM-I ratio was determined by quantifying the signal intensity of the bands using Image J (National Institute of Health) software from three independent experiments.

### Immunoprecipitation and identification of a 14-kDa fragment of ChM-I

Costal cartilage was dissected out from a 4-week-old male rat. The dissected cartilage tissues (90 mg, wet weight) were frozen in liquid nitrogen, crashed by Cryopress (CP-100, Microtec Nition, Chiba, Japan), homogenized in 8 M urea extraction buffer [50 mM Tris-HCl (pH 8.0) and 1 mM EDTA], and incubated at 4°C for 2 h. The resultant tissue extracts were centrifuged to remove debris and then protein concentration was determined using BCA Protein Assay Kit (Thermo SCIENTIFIC). Three hundred microgram of protein in the cartilage extracts was incubated with 50 μg of hCHM-05 ChM-I MoAb in the immunoprecipitation buffer [1% Igepal CA-630 (Sigma), 50 mM Tris-HCl (pH 7.5), 150 mM NaCl, 1 mM EDTA, 1 mM phenylmethylsulfonyl fluoride (Sigma), 1 μg/ml leupeptin (Calbiochem, La Jolla, CA), 1 μg/ml aprotinin (Sigma), 1 μg/ml pepstatinA (Sigma)] at 4°C for 2 h. Four hundred microliter of a 50% suspension of protein G sepharose beads (GE Healthcare Bio-Sciences, Uppsala, Sweden) were then added to the mixture and incubated at 4°C for another 2 h with gentle rocking. After incubation, the beads were washed twice with the immunoprecipitation buffer and then with ice-cold PBS twice. The bound protein was eluted with 300 μl of 1× SDS sample buffer [50 mM Tris-HCl (pH 6.8), 2% SDS, 50 mM DTT, and 10% glycerol] and boiled for 5 min. The eluate was concentrated by acetone precipitation, separated by SDS-PAGE in a 12% gel, and transferred onto Immobilon-P transfer membrane (Millipore, Bedford, MA). The membranes were then stained with Coomassie Brilliant Blue R-250 for 5 min, washed with 45% methanol containing 7% acetic acid for 15 min, rinsed with distilled water three times, and rinsed with 90% methanol containing 7% acetic acid for 40 sec. The visible 14-kDa band was excised out with razor blade and was subjected to protein sequencing. The N-terminal sequence of 14-kDa protein was determined with Procise 494 HT Protein Sequencing System (ABI, Foster City, CA).

### Migration assay of HUVECs

Migration of HUVECs was evaluated by a modified Boyden chamber assay as described previously [8]. Briefly, HUVECs were grown to subconfluence and serum-starved for the last 4 h in αMEM containing 0.5% FBS. The cells were then resuspended in αMEM containing 0.1% BSA (7×10^4^ cells/200 μl), preincubated with G-rhChM-I or ΔN-ChM-I for 30 min prior to seeding onto the vitronectin-coated cell culture inserts (8 μm-sized pore filter, BD Biosciences). Chemotactic migration of cells was induced by the addition of 20 ng/ml recombinant human VEGF-A_165_ (R&D Systems, Minneapolis, MN) in the lower chamber (600 μl), and the cells were allowed to migrate for 4 h at 37°C. The number of cells that had migrated to the undersurface of the insert was counted in five representative fields at a higher magnification (×200 magnification) per insert.

### Preparation of frozen sections and immunohistochemistry

Legs of mouse embryos at embryonic day 16.5 (E16.5) were fixed in 4% paraformaldehyde (PFA) in PBS at 4°C for 3 h, infiltrated with 18% sucrose/PBS, embedded in O.C.T. compound (Sakura Finetek, Tokyo, Japan), frozen in liquid nitrogen, and sectioned at a thickness of 8 μm using a disposable stainless-steel blade (Leica Microsystems, Wetzlar, Germany). Sections were fixed in 4% PFA/PBS for 5 min and decalcified with 0.25 M EDTA/PBS at room temperature for 60 min. After permeabilization with 0.25% Triton X-100/PBS for 5 min, sections were incubated with testicular hyaluronidase (Sigma) at 37°C, at 200 U/ml for 10 min. After fixation in 4% PFA/PBS for 3 min, sections were pretreated with blocking reagents and then incubated with primary antibodies. For immunostaining with hCHM-05 ChM-I MoAb, sections were pretreated with the blocking reagents of M.O.M. Immunodetection Kit (VECTOR Laboratories, Inc. Burlingame, CA) and incubated with hCHM-05 ChM-I MoAb (diluted 1∶500) at room temperature for 30 min. Specificity of the antibody was confirmed by the absorption test done in the presence or absence of G-rhChM-I. For absorption of hCHM-05 ChM-I MoAb, 2 μg of the antibody was incubated with 1.5 nmol of G-rhChM-I in 1% BSA/PBS overnight. For immunostaining with N-ChM-I Ab (diluted 1∶1000) or anti-CD31 antibody (diluted 1∶2000), sections were pretreated with 3.2% skim milk/PBS at 4°C for 10 min, and incubated overnight with each antibody at 4°C. Sections were then incubated with an appropriate secondary antibody labeled with Alexa fluor 488 or Alexa fluor 594 (diluted 1∶500; Invitrogen). Cell nuclei were counterstained with 4′, 6-diamino-2-phenylindole (DAPI) (diluted 1∶1000; Sigma). For immunostaining of MMP9 (diluted 1∶200) and MMP13 (diluted 1∶500), sections were pretreated in sodium citrate buffer (pH 6) at 100°C for 10 min and processed for immunostaining as described above. The sections labeled with fluorescent molecules were mounted in ProLong Antifade reagent (Invitrogen) for microscopic observation. A Leica DMRXA microscope and a Leica DC500 (Leica Microsystems) were used for capturing the images.
